# Telephones and Improved Hearing

**Published:** 1884-05

**Authors:** 


### TELEPHONES AND IMPROVED HEARING.

IT Appears, says the New Haven *Register*, that many people who have telephones in their houses or places of business, and use them frequently, find their hearing bettered. The best testimony however, comes from the central office: At each switch-board sits an operator, generally a girl, who, from morning till night haggles with unreasonable subscribers till her head fairly rings with ' hello," " all right," " go ahead." Now her ear is drilled to catch the faintest sound. If an operator were to take a switch-board one day in the week, only, and do all the work required on that day the practice would doubtless be detrimental, because it would be exhaustive to both the muscular and nervous make-up of the ear. Systematic use of the telephone seems to develop the hearing above its normal acuteness. The difficulty which people find in working the telephone comes from inability to fix the attention on what is heard.

